# KIOM-79, an Inhibitor of AGEs–Protein Cross-linking, Prevents Progression of Nephropathy in Zucker Diabetic Fatty Rats

**DOI:** 10.1093/ecam/nep078

**Published:** 2011-06-23

**Authors:** Young Sook Kim, Junghyun Kim, Chan-Sik Kim, Eun Jin Sohn, Yun Mi Lee, Il-Ha Jeong, Hyojun Kim, Dae Sik Jang, Jin Sook Kim

**Affiliations:** Diabetic Complications Research Center, Division of Traditional Korean Medicine (TKM) Integrated Research, Korea Institute of Oriental Medicine, Daejeon, Republic of Korea

## Abstract

Advanced glycation end products (AGEs) have been implicated in the development of diabetic complications, including diabetic nephropathy. KIOM-79, an 80% ethanolic extract obtained from parched Puerariae Radix, gingered Magnolia Cortex, Glycyrrhiza Radix and Euphorbia Radix, was investigated for its effects on the development of renal disease in Zucker diabetic fatty rats, an animal model of type 2 diabetes. *In vitro* inhibitory effect of KIOM-79 on AGEs cross-linking was examined by enzyme-linked immunosorbent assay (ELISA). KIOM-79 (50 mg/kg/day) was given to Zucker diabetic fatty rats for 13 weeks. Body and kidney weight, blood glucose, glycated hemoglobin, urinary albumin and creatinine excretions were monitored. Kidney histopathology, collagen accumulation, fibrinogen and transforming growth factor-beta 1 (TGF-**β**1) expression were also examined. KIOM-79 reduced blood glucose, kidney weight, histologic renal damage and albuminuria in Zucker diabetic fatty rats. KIOM-79 prevented glomerulosclerosis, tubular degeneration, collagen deposition and podocyte apoptosis. In the renal cortex, TGF-**β**1, fibronectin mRNA and protein were significantly reduced by KIOM-79 treatment. KIOM-79 reduces AGEs accumulation *in vivo*, AGE–protein cross-linking and protein oxidation. KIOM-79 could be beneficial in preventing the progression of diabetic glomerularsclerosis in type 2 diabetic rats by attenuating AGEs deposition in the glomeruli.

## 1. Introduction

Chronic hyperglycemia is a common feature of all forms of diabetes mellitus and accelerates non-enzymatic browning in the Maillard reaction between reducing sugars and free reactive amino groups of proteins. The irreversible formation of advanced glycation/lipoxidation end products (AGEs/ALEs) affects proteins and lipids such as hemoglobin, collagen and lipoprotein and causes damage to the kidney, eyes and blood vessels [1, 2]. Diabetic nephropathy is one of the main causes of end-stage renal disease and is characterized by proteinuria, progressive accumulation of glomerular extracellular matrix (ECM) and glomerulosclerosis. The AGEs inhibitors or cross-link breakers such as aminoguanidine (AG), pyridoxamine, LR-90 and ALT-711 (alagebrium), have been reported to attenuate various functional and structural manifestations of diabetic microvascular disease within the kidney in experimental animals [3–5]. There is no Food and Drug Administration-approved agents for the specific indication of AGEs modification to date, although these synthetic and natural compounds are in clinical and preclinical testing [2]. The Zucker fatty rats, a widely studied model of obesity and insulin resistance, exhibit glomerular hypertrophy, thickening of basement membranes and diffuse expansion of the glomerular mesangial matrix that resemble some of the histologic changes seen in diabetic nephropathy with proteinuria [6, 7].

Many traditional medicinal herbs have been used widely for the treatment of diabetes and diabetic complications in Asian countries for hundreds of years, and are still in present use [8, 9]. In the past 5 years, extracts or single compounds from herbs have been screened for possible AGEs inhibitory or breaking effects using various fluorescence and immunological techniques in our laboratory. KIOM-79 is composed of four herbal medicines, which are parched Puerariae Radix, gingered Magnoliae Cortex, Glycyrrhizae Radix and Euphorbiae Radix [10]. Previous studies suggested that KIOM-79 possesses stronger inhibitory activity against AGEs formation *in vitro* than the individual herbs or AG. It also shows pharmacological effects on diabetic complications in a non-obese rat model [11, 12]. Prevention of the initiation of nephropathy or retardation of the progression of glomerulosclerosis is important key for the development of a therapeutic agent for renal disease. The aim of this study was to examine the effect of KIOM-79, a new herbal inhibitor of AGEs-protein cross-linking, on the progression of diabetic nephropathy in Zucker diabetic fatty rats. KIOM-79 demonstrated a protective effect as measured by renal functional and structural parameters, including TGF-*β*1 and the extracellular matrix proteins, such as fibronectin and collagen. We also found an anti-apoptotic effect of KIOM-79 in glomerular podocytes.

## 2. Methods

### 2.1. Materials

Cortex of *Magnolia officinalis* Rehd. et Wils., Radix of *Pueraria lobata* Ohwi, Radix of *Glycyrrhiza uralensis* Fisch and Radix of *Euphorbia pekinensis* Ruprecht were identified by botanist, Prof. J.H. Kim (Division of Life Science, Daejeon University, Korea). These medicinal herbs that constitute KIOM-79 were prepared as previously described [10, 11]. The quality of KIOM-79 was controlled by HPLC [11]. Biochemical reagents were from Sigma (St. Louis, MO, USA) and antibodies from Cell Signaling or Santa Cruz, unless otherwise indicated. All other chemicals were of analytical grade.

### 2.2. In vitro Assay for Ages Inhibition/Breaking

For the AGEs inhibition assay, AGE-BSA was incubated in either the presence or absence of KIOM-79 or AG in collagen-coated 96-well plates [13]. Collagen-AGE-BSA cross-linking was detected using rabbit-anti-BSA antibody, horseradish peroxidase-linked goat-anti rabbit IgG antibody and H_2_O_2_ substrate containing ABTS chromogen. Inhibition of collagen-AGE-BSA cross-linking was expressed as the percentage decrease in optical density (OD = 410 nm). We calculated the IC_50_ concentration (*μ*g/ml) as 50% inhibition of collagen-AGE-BSA cross-linking. For the breaking assay, AGE-BSA was pre-incubated on collagen-coated 96-well plates for 24 h and the collagen-AGE-BSA complexes were incubated in either the presence or absence of KIOM-79 or ATL-711 [14]. Breaking levels were measured as the percentage decrease in optical density (OD = 410 nm). ALT-711 was used as a positive control for AGEs cross-link breaker.

### 2.3. Animals and Experimental Design

Male Zucker fatty (*fa/fa*, ZF) and Zucker lean (*fa/+* or +/+, ZL) rats were obtained at 6 weeks of age from Charles River Laboratory (Wilmington, MA, USA). Animals were divided into four groups: Zucker lean rats (ZL, *n* = 7); Zucker fatty rats (ZF, *n* = 7); Zucker fatty rats treated with AG (50 mg/kg body weight, ZF + AG, *n* = 7); Zucker fatty rats treated with KIOM-79 (50 mg/kg body weight, ZF + KIOM-79, *n* = 8). Rats were allowed free access to water and food for 13 weeks and, at 3 weeks intervals, their intake of water and food for a 24-h period was measured. All experimental protocols for animal care involving the use of animals were conducted in accordance with National Institutes of Health (NIH) Guidelines and approved by the Committee on Animal Care of our institute.

### 2.4. Analysis of Metabolic Data

When the rats reached 20 weeks of age, blood glucose, HbA1c (A1C), serum creatinine, total cholesterol, triglycerides, HDL, LDL and free fatty acid were measured using an automated analyzer (Wako, Japan). Blood samples were collected from the tail vein after a 16-h fast. Individual rats were placed in metabolic cages to obtain 24-h urine collections and daily urinary albumin excretion levels were measured.

### 2.5. Morphological Studies

Renal cortexes were fixed in 10% formaldehyde and embedded in paraffin, and 4-*μ*m thick sections were prepared. The sections were stained with periodic acid-Schiff (PAS) reagent and hematoxylin as a counterstain. Glomerular tuft and mesangial matrix areas were measured using image analysis NIH Image J software (National Institutes of Health, Bethesda, MD, USA). The cross-section yielding the maximum diameter of the glomerulus was photographed and converted into a digital image. A total of 40 glomeruli were randomly chosen from each rat kidney. To determine collagen deposition in the kidneys, paraffin sections were deparaffinized, sectioned and stained using Masson's trichrome. For AGEs immnohistochemistry, the deparaffinized sections were hydrated and treated with 1% H_2_O_2_ in methanol. Sections were incubated with anti-AGEs antibody (1 : 100, Transgenic Inc. Kobe, Japan) for 2 h at room temperature using a standard manual immunoperoxidase procedure with streptavidin-peroxidase (LSAB 2 kit, Dako, CA, USA). The TUNEL assay was carried out according to the manufacturer's instructions (Roche Diagnostics, Meylan, France). Kidney sections stained by immunofluorescence of synaptopodin (1 : 250) and Wilms tumor antigen-1 (WT-1, 1 : 250) were observed by fluorescence microscopy (Olympus BX51) equipped with an Olympus DP 70 camera.

### 2.6. RNA Extraction and RT-PCR

Total RNA isolation and RT-PCR were as previously described [15]. For RT-PCR, cDNA was synthesized with 3 *μ*g of RNA using RT-primix (Bioneer, Korea). The upstream and downstream primers for rat TGF-*β*1 mRNA were 5′-CGA GGT GAC CTG GGC ACC ATC CAT GAC-3′ and 5′-CTG CTC CAC CTT GGG CTT GCG ACC CAC-3′, yielding a 409-bp product. *β*-Actin was used as an internal control, 5′-CGT AAA GAC CTC TAT GCC AA-3′ and 5′-AGC CAT GCC AAA TGT GTC AT-3′, yielding a 350-bp product. The RT-PCR products were separated by electrophoresis and DNA band intensities in agarose gels and quantitated with densitometry (Las-3000, Fuji photo, Tokyo, Japan).

### 2.7. Western Blot Analysis

Western blot was performed using a previously described method [15]. Renal cortex were lysed in solutions containing 250 mM sucrose, 1 mM ethylenediaminetetraacetic acid (EDTA), 0.1 mM phenylmethylsulfonyl fluoride (PMSF) and 20 mM potassium phosphate buffer, at pH 7.6 with a homogenizer at 3000 rpm. Equal amounts of protein (50 *μ*g/lane) were subjected to immunoblotting with the indicated antibodies. The antibodies used were TGF-*β*1 and fibronectin (1 : 1000, Santa Cruz Biotechnology, CA, USA). The bound horseradish peroxidase-conjugated secondary antibody was detected using an enhanced chemiluminescence detection system (iNtRON Biotechnology, Korea). Protein expression levels were determined by analyzing the signals captured on the nitrocellulose membranes using an image analyzer (Las-3000, Fuji photo, Tokyo, Japan).

### 2.8. Statistical Analysis

Data are expressed as mean ± SD and analyzed by one-way analysis of variance (ANOVA) followed by Tukey's multiple comparison test or by unpaired Student's *t*-test using GraphPad *Prism 4.0* software (Graph pad, San Diego, CA, USA). Differences with a value of *P* < .05 were considered statistically significant.

## 3. Results

### 3.1. Inhibitory Effect of KIOM-79 on AGE-BSA and Collagen Cross-Linking In Vitro

**Figure 1:**
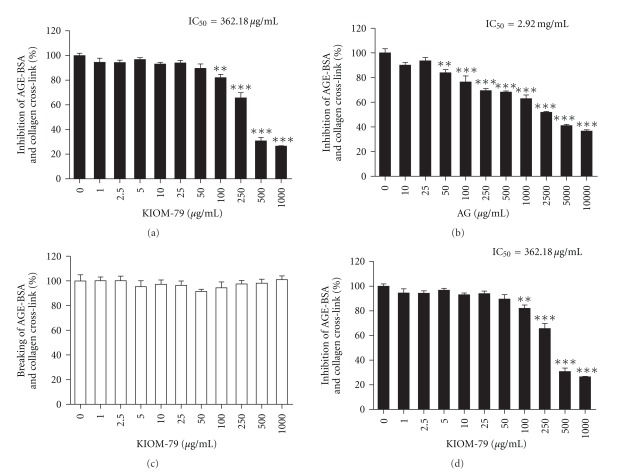
Inhibitory effect of KIOM-79 on AGE-BSA and collagen cross-linking *in vitro*. Inhibition of KIOM-79 (a) and AG (b) on AGE-BSA and collagen cross-linking. Breaking of KIOM-79 (c) and ALT-711 (d) on AGE-BSA and collagen cross-links. ***P < .001; **P < .01 versus untreated group, respectively.

To investigate whether KIOM-79 could inhibit or break the AGEs cross-link, AGE-BSA was incubated or pre-incubated with KIOM-79 in collagen-coated plates. KIOM-79 (IC_50_ = 362.18 *μ*g/ml) exhibited much stronger inhibitory activity on AGE-BSA binding with collagen than AG (IC_50_ = 2.92 mg/ml), a well-known glycation inhibitor (Figures 1(a) and 1(b)). However, KIOM-79 did not affect AGEs breaking on the binding of AGE-BSA and collagen (Figure 1(c)).  Figure 1(d) shows the breaking effect of ALT-711 (IC_50_ = 16.50 mg/ml), an AGEs cross-link breaker.

### 3.2. Body Weight, Kidney Weight and Metabolic Parameters in Blood

**Figure 2:**
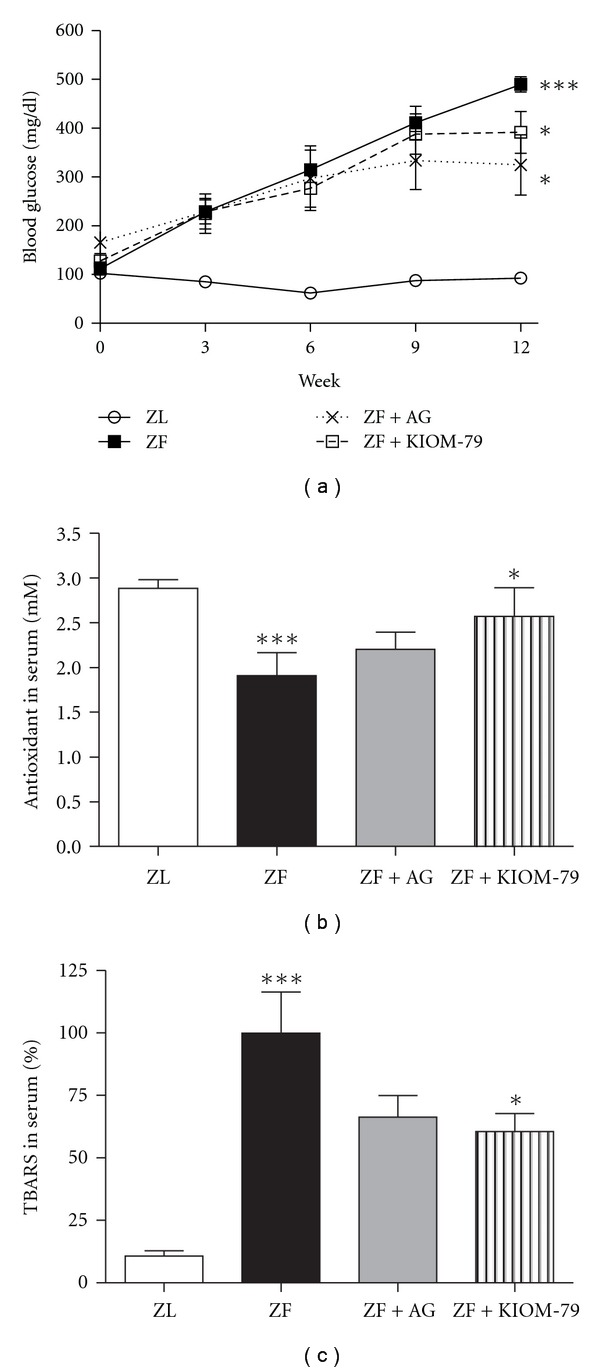
Blood glucose levels and antioxidant effect of KIOM-79 in serum of ZF rats. (a) Blood glucose; (b) antioxidant effect in serum; (c) TBARS levels in serum. ZL, normal lean rat; ZF, ZDF rat; ZF + AG, ZDF rat treated with AG (50 mg/kg); ZF + KIOM-79, ZDF rat treated with KIOM-79 (50 mg/kg). All data are expressed as mean ± SD (*n* = 6, resp.). ***P < .001 versus ZL group; *P < .05 versus ZF group.

In ZF rats at 20 weeks of age, body weight was increased compared with ZL and did not change compared to rats that did not receive AG or KIOM-79 treatment. Kidney weight was increased in ZF rats and reduced by KIOM-79 treatment (Table 1). Blood glucose, LDL, HDL, TG, total cholesterol and HbA1c levels were significantly increased in ZF rats (*P* < .001 versus control group). ZF rats treated with AG or KIOM-79 showed significant reduction in blood glucose, LDL, TG and HbA1c as compared with untreated ZF rats (Figure 2(a) and Table 2). However, no differences in levels of liver enzymes, such as aspartate aminotransferase (AST) and alanine aminotransferase (ALT), were noted between treated and untreated ZF rats.

To determine the antioxidant effect of KIOM-79 in serum, antioxidant activity and malondialdehyde (MDA) levels were tested. In ZF rats, the antioxidant levels significantly decreased (*P* < .001) and MDA levels increased (Figures 2(b) and 2(c)). KIOM-79 treatment significantly increased the reduced antioxidant enzyme activity and decreased the elevated MDA levels.

### 3.3. Morphology and Renal Function

**Figure 3:**
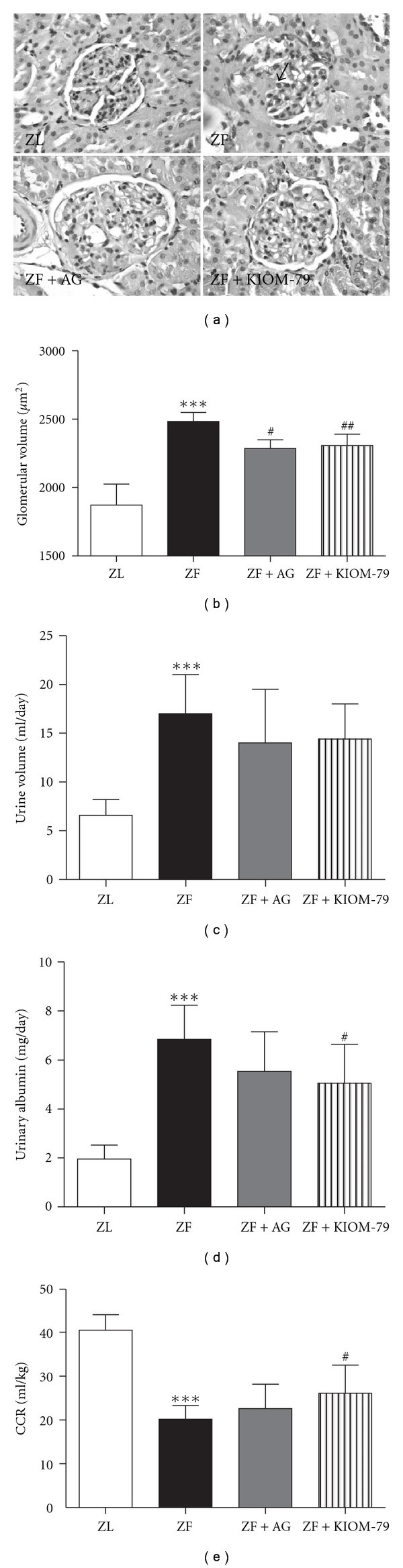
Renal histopathology and function. (a) PAS staining of glomeruli. ×400; (b) glomerular volume in each group. Urine volume (c), albuminuria (d) and creatinine clearance (CCR) (e). ZL, normal lean rat; ZF, ZDF rat; ZF + AG, ZDF rat treated with AG (50 mg/kg); ZF + KIOM-79, ZDF rat treated with KIOM-79 (50 mg/kg). All data were expressed as mean ± SD (*n* = 6-7). ***P < .001 versus ZL; ^##^ *P* < .01; ^#^ *P* < .05 versus ZF group, respectively.

Mesangial matrix expansion is considered a hallmark of diabetic nephropathy.  Figure 3(a) suggests accelerated mesangial matrix expansion as characterized in ZF rats (arrow). Mean glomerular volume was determined by computer-assisted image analysis. ZF rats revealed a greater than 32.7% increase in glomerular volume compared to ZL rats. AG or KIOM-79 treatment significantly reduced the glomerular volume (Figure 3(b)). At 18 weeks of age, ZF rats showed focal segmental glomerularsclerosis (FSGS), tubulointerstitial damage, proteinuria and increased kidney weight in contrast to age-matched ZL rats [7]. AG and KIOM-79 treatment ameliorated glomerularsclerosis, albuminuria and creatinine clearance, compared with the untreated ZF rats (Figures 3(d) and 3(e)).

### 3.4. Expression of Collagen, Fibronectin and TGF-*β*1

**Figure 4:**
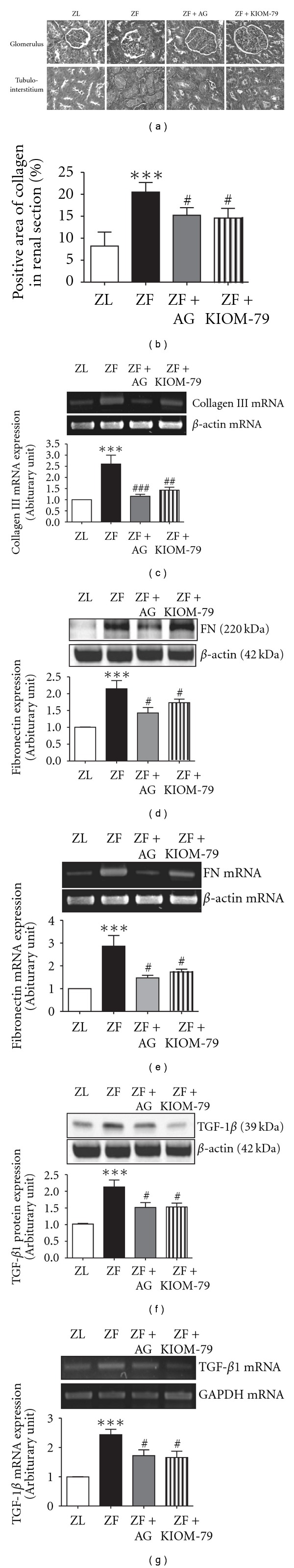
Effect of KIOM-79 on collagen accumulation and on expression of fibronectin and TGF-*β*1 in the renal cortex of ZF rats. (a) Masson's trichrome stain shows collagen accumulation in the renal cortex from normal lean rat, ZF rat, ZF rat treated with AG (50 mg/kg), and ZF rat treated with KIOM-79 (50 mg/kg). ×400. (b) Morphometric analysis of Masson's trichrome-positive areas in renal cortex in each group. The renal cortex from rats was immunoblotted using specific antibodies for fibronectin (d) and TGF-*β*1 (g). Total RNA was isolated and collagen III (c), fibronectin (f) and TGF-*β*1 (h) expression were measured by RT-PCR. All data are expressed as mean ± SD (*n* = 6, resp.).***P < .001 versus ZL group; ^###^ *P* < .001; ^##^ *P* < .01; ^#^ *P* < .05, versus ZF group, respectively.

To investigate the effect of KIOM-79 on expression of extracellular matrix protein and mRNA, we measured collagen, fibronectin and TGF-*β*1 in renal tissue using immunoassay and RT-PCR. Diabetic ZF rats were associated with an increase in collagen protein expression in the glomeruli and tubulointerstitium using Masson's trichrome (Figures 4(a) and 4(b), *P* < .001). The decrease of collagen in the glomeruli was more prominent in diabetic ZF rats treated with AG and KIOM-79 than in diabetic ZF rats (*P* < .05, Figure 4(b)). Specifically, extracellular matrix molecules, such as collagen type III and fibronectin mRNA expression were significantly decreased in diabetic ZF rats treated with AG and KIOM-79 (Figures 4(c) and 4(f)). TGF-*β*1, a key regulator of these extracellular matrix genes, has been implicated in the pathogenesis of diabetic nephropathy [16]. Expressions of TGF-*β*1 mRNA and protein were decreased in diabetic ZF rats treated with AG and KIOM-79 (Figures 4(g) and 4(h)).

### 3.5. Quantitation of AGEs

**Figure 5:**
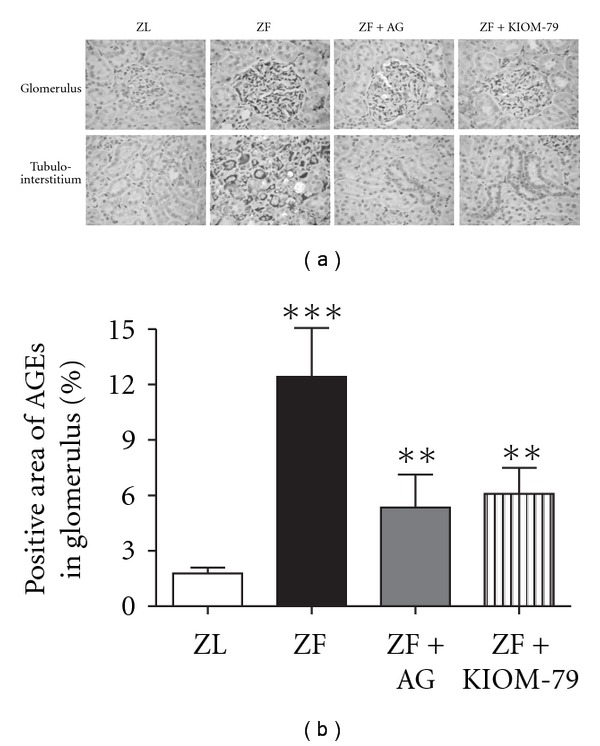
Effect of KIOM-79 on AGEs accumulation in the renal cortex of ZF rats. (a) Paraffin sections of kidney immunolabeled with AGEs from ZL and ZF rats. (b) AGEs immunostaining was quantified after 13 weeks of treatment. ***P < .001 versus ZL group; **P < .01 versus ZF group.

Immunohistochemical staining of AGEs in the glomeruli and tubulointerstitium demonstrated a significant increase in the ZF rats as compared with the ZL rats. This was attenuated by both AG and KIOM-79 (Figures 5(a) and 5(b)).

### 3.6. Anti-Apoptotic Effect of KIOM-79 in the Renal Podocytes of ZF Rats

**Figure 6:**
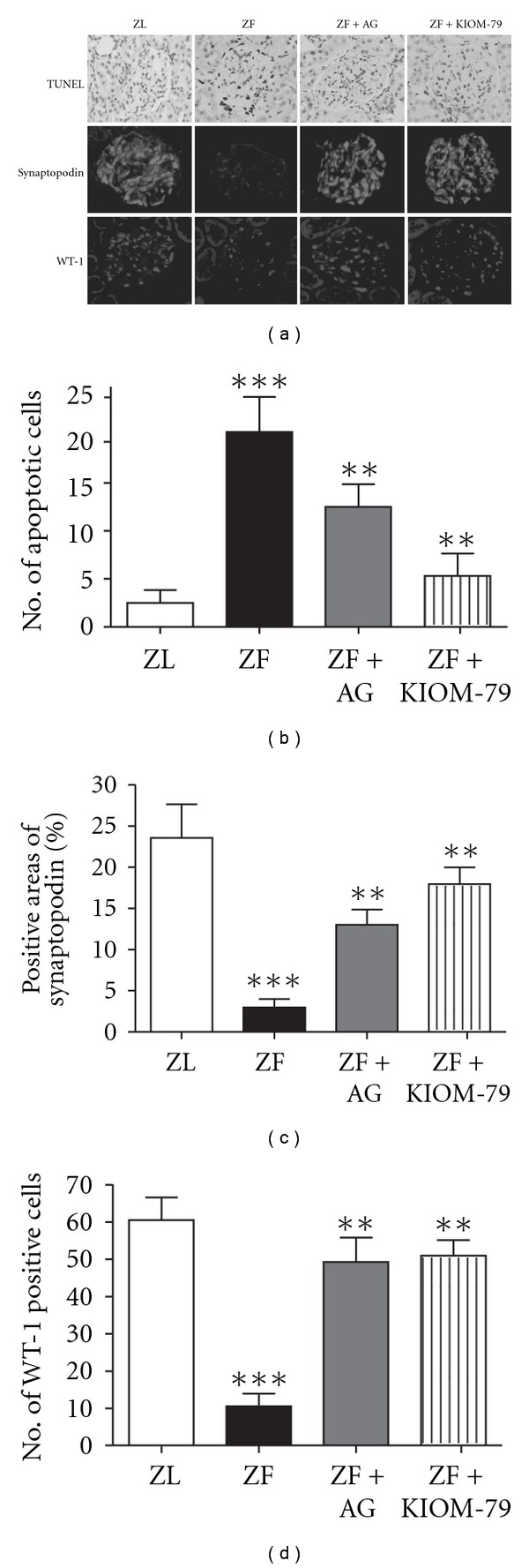
Anti-apoptotic effect of KIOM-79 in the renal podocyte of ZF rats. (a) A representative photomicrograph of TUNEL-positive cells, synaptopodin, and WT-1 in the glomerulus. Renal cortex from rats was stained using specific antibodies for synaptopodin and WT-1, which are specific markers of podocytes. Quantitative analyses of apoptotic cells (b), positive areas of synaptopodin (c) and positive cells of WT-1 (d). ***P < .001 versus ZL group; **P < .01 versus ZF group.

To determine the anti-apoptotic effect of KIOM-79, TUNEL assay was performed. In the ZF rats, TUNEL-positive cells per glomerular section were significantly increased at 13 weeks of age compared with ZL rats (Figures 6(a) and 6(b)). AG and KIOM-79 treatments were effective in reducing apoptosis in the diabetic ZF rats. Average numbers of podocytes per glomerular section were determined by counting cells and measuring areas that were positively labeled with two podocyte markers, such as synaptopodin and WT-1 [17, 18]. In ZF rats at age 20 weeks, synaptopodin and WT-1 positive cell counts tended to decrease compared with age-matched ZL rats. Treatment with AG and KIOM-79 visibly increased the positive cells and areas in the kidney glomeruli (Figures 6(a), 6(c), and 6(d)).

## 4. Discussion

KIOM-79 is an 80% ethanolic extract of four herbal medicinals, which are parched Puerariae Radix, gingered Magnoliae Cortex, Glycyrrhizae Radix and Euphorbiae Radix. These herbal medicines are used frequently for the treatment of diabetes or diabetic complications in traditional medicine in Korea and other countries [8, 9]. Our previous studies showed that KIOM-79 has anti-diabetic effects, such as protection of beta-cells, reduced glucose in non-obese type 2 diabetic rats, inhibition of AGEs formation *in vitro* and anti-inflammatory effects [10, 11, 19]. The results of this study showed that KIOM-79, an herbal inhibitor of AGEs-collagen cross-linking, reduced the development of diabetic nephropathy in the type 2 diabetic animal model, Zucker diabetic fatty rats. Based on immunohistochemical measurements, the current study confirmed that KIOM-79 prevents AGEs accumulation in the diabetic kidney and reduces hyperglycemia, apoptosis of podocyte and oxidation in renal cortex. Furthermore, KIOM-79-treated diabetic rats showed statistically significant improvement in renal functions such as albuminuria and creatinine clearance. We have demonstrated that these effects correlate with prevention of several key aspects of renal pathology in Zucker diabetic fatty rats.

A number of studies have shown that the hyperglycemia has an important role in the pathogenesis of diabetic complications by increasing protein glycation and the gradual formation of AGEs in tissues, especially the kidney. There is considerable interest in inhibitory compounds of AGEs-protein cross-link formation or breaking because of their therapeutic potential [2, 20]. Several natural and synthetic compounds have been proposed and tested as inhibitors of AGEs formation. AGEs inhibitors such as AG, pyridoxamine, ALT-946 and LR-90 have been reported to attenuate mesangial expansion and albuminuria in animal models of diabetic renal disease [4, 20–26]. Furthermore, flavonoids such as oxerutin and disomin have demonstrated a capacity to decrease the renal accumulation of AGEs and prevent apoptosis of glomerular cells in diabetic nephropathy [24, 27]. The flavonoids-induced decrease in glycation is associated with an increase in the antioxidant component [24].

In this study, KIOM-79, a natural product, inhibited AGE-BSA and collagen cross-link at 8-fold less concentration (IC_50_) than AG. However, it had no effect on the breaking of AGE-BSA and collagen cross-linking. KIOM-79-treated ZF rats had antioxidant activity in serum and low blood glucose when compared with untreated ZF rats. ALT-711, an AGEs cross-link breaker, delayed established diabetic nephropathy in db/db mice by reducing the systemic AGEs pools and facilitating the urinary excretion of AGEs [5]. AGEs levels in glomerular section were significantly reduced in KIOM-79-treated ZF rats. The administration of KIOM-79 significantly ameliorated the ratio of kidney weight to body weight in ZF rats. Kidney weight/body weight expresses as a function of body weight, glomerular hypertrophy and albuminuria in diabetic nephropathy. Furthermore, KIOM-79 had inhibitory effects on expression of TGF-*β*1 and fibronectin in renal cortex and podocyte apoptosis. We found previously that KIOM-79 scavenges intracellular reactive oxygen species (ROS) thereby preventing DNA damage. Moreover, it inhibited apoptosis of beta-cells exposed to streptozotocin via radical scavenging activity and activation of antioxidant enzymes [19]. High glucose-induced ROS promote podocyte apoptosis and AGEs accelerate podocyte injury by activation of the FOXO4 transcription factor [28]. Antioxidant therapy prevents podocyte apoptosis [2]. Our recent study showed that KIOM-79 prevented lens opacity in xylose-induced lens through inhibition of aldose reductase and reduction of reduced glutathione (GSH) [29]. Furthermore, KIOM-79 prevents cataracts, apoptosis in neuronal cells of the retina and ameliorates the development of diabetic retinopathy in animal models of type 2 diabetic [30].

AGEs inhibitors are nucleophilic compounds designed to trap reactive carbonyl or dicarbonyl intermediates in AGEs formation and have potent chelating activity, making it difficult to dissect the antioxidative effects [25]. The mechanism of action of KIOM-79 *in vivo* is still is not clear. However, KIOM-79 is composed of natural products and has been used widely for the treatment of diabetes. Based on our findings, the inhibition of AGEs cross-linking in the kidney and the antioxidant effect on podocyte apoptosis by KIOM-79 might ameliorate diabetic nephropathy and prevent the progression to end-stage renal failure. Furthermore, these data support the premise that KIOM-79 is effective for treatment for diabetic complications due to inhibition of AGEs accumulation in the kidney.

In summary, this study showed that KIOM-79 is more potent than previously used synthetic compound (such as AG) on inhibition of AGE-protein cross-linking and modulates the toxic effects of AGEs in type 2 diabetic rats. We speculate that KIOM-79 inhibits AGEs accumulation in the renal cortex by direct or indirect interaction with AGEs-protein cross-links. KIOM-79 could be an effective treatment for diabetic nephropathy and possibly other complications.

## Funding

Grant [L08010] from the Korea Institute of Oriental Medicine.

## Figures and Tables

**Table 1:** Body weight and kidney weight of experimental rats.

	ZL	ZF	ZF + AG	ZF + KIOM-79
*Body weight (g)*				
Initial	202.0 ± 24.4	276.6 ± 20.3	278.4 ± 9.1	266.5 ± 18.4
Final	338.5 ± 40.5	433.2 ± 69.4***	421.4 ± 66.1	414.6 ± 45.0
*Kidney (mg/100 g body weight)*				
Left	280.9 ± 2.7	341.1 ± 19.0***	343.7 ± 26.8	316.6 ± 17.1*
Right	302.6 ± 8.2	348.5 ± 25.9***	333.7 ± 26.2	320.1 ± 17.8*

ZL: normal lean rat; ZF: ZDF rat; ZF + AG: ZDF rat treated with AG (50 mg/kg); ZF + KIOM-79: ZDF rat treated with KIOM-79 (50 mg/kg). All data were expressed as mean ± SD.

***P < .001 versus ZL group; *P < .05 versus ZF group.

**Table 2:** Plasma chemistry of experimental rats.

	ZL	ZF	ZF + AG	ZF + KIOM-79
AST (U/l)	121.79 ± 16.10	177.70 ± 49.86	124.03 ± 16.09	137.61 ± 34.66
ALT (U/l)	48.49 ± 6.78	116.07 ± 17.23***	89.60 ± 26.12	105.76 ± 54.35
Total cholesterol (mg/dl)	100.61 ± 7.27	254.72 ± 18.13***	229.28 ± 43.65	228.33 ± 21.69
HDL-Chol (mg/dl)	34.59 ± 2.13	72.72 ± 6.88***	70.53 ± 15.77	68.24 ± 10.32
LDL-Chol (mg/dl)	8.64 ± 0.81	34.63 ± 6.47***	26.98 ± 9.84*	26.27 ± 7.88*
Triglyceride (mg/dl)	93.09 ± 24.10	1112.60 ± 330.70***	817.12 ± 294.08*	881.04 ± 335.54*
FFA (uEq/l)	896.29 ± 185.25	1775.83 ± 331.57	1677.67 ± 239.19	1224.14 ± 172.37**
HbA1c (%)	3.71 ± 0.11	7.78 ± 1.19***	6.94 ± 1.07*	7.07 ± 0.61*

ZL: normal lean rat; ZF: ZDF rat; ZF + AG: ZDF rat treated with AG (50 mg/ml); ZF + KIOM-79: ZDF rat treated with KIOM-79 (50 mg/kg). All data were expressed as mean ± SD.

***P < .001 versus ZL group; *P < .05 versus ZF group; **P < .01 versus ZF group.
